# Patient characteristics and outcome of CD20-positive HIV-associated lymphoma: a single-center KwaZulu-Natal, South African hospital 12-year retrospective review

**DOI:** 10.1186/s43046-022-00131-6

**Published:** 2022-08-01

**Authors:** Nadine Rapiti, Nada Abdelatif, Anand Rapiti, Mahomed-Yunus Moosa

**Affiliations:** 1 Department of Haematology, NHLS/University of KwaZulu Natal/King Edward VIII Hospital, Durban, South Africa; 2 Biostatistics Research Unit, South African Medical Research Council, Durban, South Africa; 3 Department of Neurosurgery, Inkosi Albert Luthuli Central Hospital, Durban, South Africa; 4 Department of Infectious Diseases, University of KwaZulu-Natal, Durban, South Africa

**Keywords:** HIV-associated lymphoma, CD20-positive lymphoma, AIDs-related lymphoma, Rituximab

## Abstract

**Background:**

Due to the high prevalence of HIV, HIV-associated lymphoma (HAL) is a common malignancy in South Africa. However, there is a paucity of literature on HAL from this region. The objective of this study was to profile the clinical characteristics and outcome of CD20-positive HAL treated with cyclophosphamide, doxorubicin, vincristine, and prednisone (CHOP), with or without rituximab (R), from a single center in KwaZulu -Natal, South Africa.

**Methods:**

Retrospective chart review of adult patients treated from 2006 to 2018 for HIV-associated CD20-positive lymphoma. The clinical characteristics, complete response (CR), and 2-year overall survival (OS) are described.

**Results:**

The analysis included 102 patients, 54% females, median age of 39 years, and median CD4 cell count of 196 cells/μL. Bone marrow involvement was noted in 5%. Eighty-six percent of the cohort received concomitant antiretroviral therapy and chemotherapy, 76% of the CHOP group, and 92% of the R-CHOP group. Overall, a CR was seen in 55% (95% CI 45%; 65%), with a 2-year OS of 59% (95% CI 50%, 69%). A CR was attained in 46% on CHOP and 64% on R-CHOP, with a 2-year disease-free survival (DFS) for CHOP of 42% and 50% for R-CHOP.

**Conclusion:**

Although the clinical characteristics and laboratory findings are similar to other higher-income cohorts, there was a difference in gender and incidence of marrow involvement. The low incidence of marrow involvement has prompted more routine use of immunohistochemistry and flow cytometry in staging marrows of HAL locally. Further randomized studies are required for the establishment of locally validated, cost-effective treatment guidelines.

## Background

Worldwide, HIV-associated lymphoma (HAL) is a common HIV-related malignancy [1]. Most are aggressive, high-grade B cell malignancies and are classified as AIDS-related lymphomas [2]. Prior to antiretroviral therapy (ART), the incidence was 60–200-fold higher than that seen in HIV-negative subjects, but has decreased to 11–25-fold with the widespread use of ART [3]. The prevalence of HIV in South Africa (SA) is estimated at 13.5% (8 million people), with the province of KwaZulu-Natal leading the provinces at a seroprevalence of 18% [4, 5].

Treatment for HAL has evolved from low dose chemotherapy to full dose cyclophosphamide, doxorubicin, vincristine, prednisone (CHOP) with ART [6–8]. Rituximab (R), a chimeric anti-CD20 monoclonal antibody, was initially added to the treatment of CD20-positive lymphomas in HIV-negative patients after randomized controlled trials showed improved complete response (CR) and improved event-free and overall survival with the addition of rituximab [9]. A meta-analysis by Barta et al., using individual patient data of 1546 patients with HAL, from 19 clinical trials from North America and Europe, demonstrated a clear survival benefit with the addition of rituximab to standard chemotherapy [10]. This ushered in R-CHOP as the regimen of choice for CD20-positive HAL [11–13].

There is limited data from South Africa on HAL, particularly on outcomes with rituximab [14]. Here we describe the presentation and outcomes of CD20-positive HAL from a resource-constrained, single institute, at the epicenter of the HIV AIDS epidemic. A shift in treatment midway through the study period, from CHOP to R-CHOP, provided an opportunity to describe outcomes with and without rituximab.

## Methods

### Clinical setting

King Edward VIII Hospital is a tertiary government hospital and services a population of 11.3 million [5]. The hematology clinic attends to approximately 600 in-patients and out-patients and 30 new referrals per month. All indigent patients with HAL, except Burkitt lymphoma, are managed here.

### Study design

A retrospective chart review was performed on all patients with CD20-positive HAL who met inclusion criteria and were started on treatment at the hematology clinic between January 2006 and December 2016. All patients were followed up to December 2018, to capture at least 2 years of survival data. Clinical information was obtained from patient charts, and laboratory data was obtained from the National Health Laboratory Service database. The study protocol was approved by the Biomedical Research Ethics Committee (BE043/17) and complied with the principles of the Declaration of Helsinki.

### Patient selection

Chart review was restricted to HIV-positive patients over the age of 12 years, with histologically proven CD20-positive HAL. Burkitt lymphoma was excluded from this analysis. Patients with misplaced clinical notes, untraceable HIV test results, or missing histology results were excluded from the analysis. For inclusion, patients had to have received at least one cycle of CHOP or R-CHOP.

### Patient diagnosis, investigation, and staging

Tissue biopsies were examined by certified histopathologists and reporting followed World Health Organization guidelines [15, 16]. All patients had Eastern Cooperative Oncology Group performance status assessments and the International Prognostic Index was calculated [17]. Clinical workup included a unilateral staging bone marrow biopsy. Cardiac function was assessed by a multiple-gated acquisition scan. Baseline staging radiology included a computerized tomography (CT) scan or a positron emission tomography (PET)-CT scan, and the Cotswolds-modified Ann Arbor system was used to stage disease [18].

### Patient treatment

All patients were offered ART at time of diagnosis of lymphoma as per national guidelines [19]. Chemotherapy for lymphoma comprised one of two regimens: intravenous cyclophosphamide, doxorubicin, vincristine, and oral prednisone (CHOP), or CHOP with rituximab (R-CHOP) [20]. Regimens were designed to be repeated every 21 days for a total of six to eight cycles. From 2006 to 2013, CHOP chemotherapy was the standard of care. When rituximab became available in 2013 all patients were offered R-CHOP as the standard of care. Patients not achieving a complete response (CR) with first-line chemotherapy, were offered salvage chemotherapy and/or radiotherapy.

Central nervous system (CNS) prophylaxis was not routinely practiced. CNS involvement was assessed clinically. If suspected, confirmation was by cerebrospinal (CSF) cytology or flow cytometry. CNS involvement was managed with alternate day intra-thecal (IT) methotrexate, cytarabine, and hydrocortisone until two consecutive CSF evaluations showed the absence of malignant cells [21]. Patients were then offered a further two to four cycles of high-dose intravenous methotrexate in addition to completing the remaining cycles of CHOP or R-CHOP chemotherapy based on willingness to be admitted for the former [22].

Neutropenia was managed with growth factors as secondary prophylaxis [23]. Patients receiving rituximab were offered isoniazid as treatment of latent tuberculosis (TB).

### Response to treatment

After three to four cycles of chemotherapy, a mid-cycle PET-CT or staging CT scan was performed. PET-CT scans were reported consistently at a single center using the Deauville scoring system [24]. Response was assessed by CT scans using the revised evaluation criteria in solid tumors to define CR, partial response, and progressive disease [25]. For a complete response (CR), the PET-CT or CT scan had to show the absence of metabolic or radiological evidence of disease respectively, either halfway through chemotherapy or at the end of treatment. On PET-CT a Deaville score of < 3 was classified as a CR. A partial response was defined as a ≥ 50% reduction in the tumor size. Relapsed or progressive disease was a ≥ 50 increase in tumor or the appearance of new lesions. Stable disease was a response not meeting any of the aforementioned criteria.

Bone marrow biopsy was only repeated if the initial biopsy showed infiltration by lymphoma. If the mid-cycle staging showed a CR, patients received a further 3–5 cycles of chemotherapy. If a mid-cycle PR was noted, patients underwent repeat radiological staging at the end of 6–8 cycles [26]. Patients with CR were reviewed quarterly. Patients with no response or progressive disease at mid-cycle or with partial or no response at the end of 8 cycles were offered second-line salvage therapy.

### Outcome

Study outcomes included CR, 2-year disease-free survival (DFS), and 2-year overall survival (OS). CR was defined as the absence of clinical and radiological evidence of lymphoma. Two-year DFS was the proportion free of disease at 2 years post-chemotherapy. Two-year OS was the proportion alive at 2 years post first hematology clinic presentation. As the cohort was an intent to treat group, the patients who demised or defaulted follow-up during the study were considered non-responders in the analysis.

### Statistical analysis

The baseline characteristics, and responses to treatment, were compared using means (with standard deviations) for normally distributed variables, medians (with interquartile ranges and ranges) for variables that were skewed, and frequencies (with percentages) for categorical variables. To determine statistically significant differences between the arms, the chi-square test, proportion test, quantile regression, and the *t* test were used for categorical and binary variables, medians, and means, respectively. For the study outcomes (DFS and OS), Kaplan-Meier curves are presented by treatment group. A *p* value less than 0.05 was considered statistically significant. Ninety-five percent confidence intervals (CIs) were constructed for the main outcomes. All analyses were done using Stata version 15.1 (Statacorp, 2015).

## Results

### Baseline characteristics

From 2006 to 2018, 129 patients with CD 20 positive HAL were seen. Twenty-seven were excluded; thirteen due to untraceable histology and/or HIV results, five did not receive chemotherapy, and nine received a regimen other than CHOP or R-CHOP. Of the remaining 102, 54% were female, median age of 39 years (range 21–62), and median CD4 cell count of 196 cells/μL (range 8–784). Fifty-six patients (55%) were not on ART at lymphoma diagnosis, 40 (39%) were started on ART, and 16 (16%) did not consent to ART. Overall, five patients (5%) had bone marrow involvement by lymphoma, with three of these patients receiving CHOP and two having R-CHOP. There were three failed bone marrow tests reported. The baseline profile is described in Table 1.

**Table 1:** Baseline profile of patients with CD20-positive HAL presenting to King Edward VIII Hospital from 2006 to 2018

	Total ***n*** = 102 (%)	CHOP ***n*** = 50 (%)	R-CHOP ***n*** = 52 (%)	***p*** value (CHOP vs R-CHOP)
**Histology**				0.540
**DLBCL**	70 (70%)	35 (70%)	35 (67%)	
**HGBCL**	31 (61%)	14 (28%)	17 (33%)	
**Other**	1 (2%)	1 (2%)	0	
**Median age in years (IQR)**	39 (12)	38 (9)	39 (12)	0.525
**Female**	55 (54%)	31 (62%)	24 (46%)	0.108
**ECOG ≥ 2**	88 (86%)	44 (88%)	44 (85%)	0.618
**Median CD4 in cells/μL (IQR)**	196 (209)	143 (175)	228 (211)	0.166
**CD4** ≤ **200 cells/**μL	53 (52%)	30 (60%)	23 (44%)	0.210
**CD4 > 200 cells/**μL	49 (48%)	20 (40%)	29 (56%)	
**ART at lymphoma diagnosis**	46 (45%)	18 (36%)	28 (54%)	0.021
**Extra nodal disease**	72 (71%)	36 (72%)	36 (69%)	0.711
**B symptoms**	32 (31%)	17 (34%)	15 (29%)	0.437
**Bulky disease**^a^	36 (35%)	18 (36%)	18 (35%)	0.778
**Stage III/IV disease**	46 (45%)	21 (42%)	25 (48%)	0.180
**IPI score**				0.398
**1 (low risk)**	17 (17%)	9 (18%)	8 (15%)	
**2 (low intermediate)**	31 (30%)	18 (36%)	13 (25%)	
**3 (high intermediate)**	23 (23%)	11 (22%)	12 (23%)	
**4–5 (high risk)**	30 (29%)	12 (24%)	19 (37%)	

*HAL* HIV-associated lymphoma, *DLBCL* diffuse large B cell lymphoma, *HGBCL* high-grade B cell lymphoma, *ECOG* Eastern Cooperative Oncology Group, *ART* antiretroviral treatment, *IPI* International Prognostic Index

^a^Bulky disease defined as tumor measuring ≥10 cm

### Treatment and outcomes

**Fig. 1:**
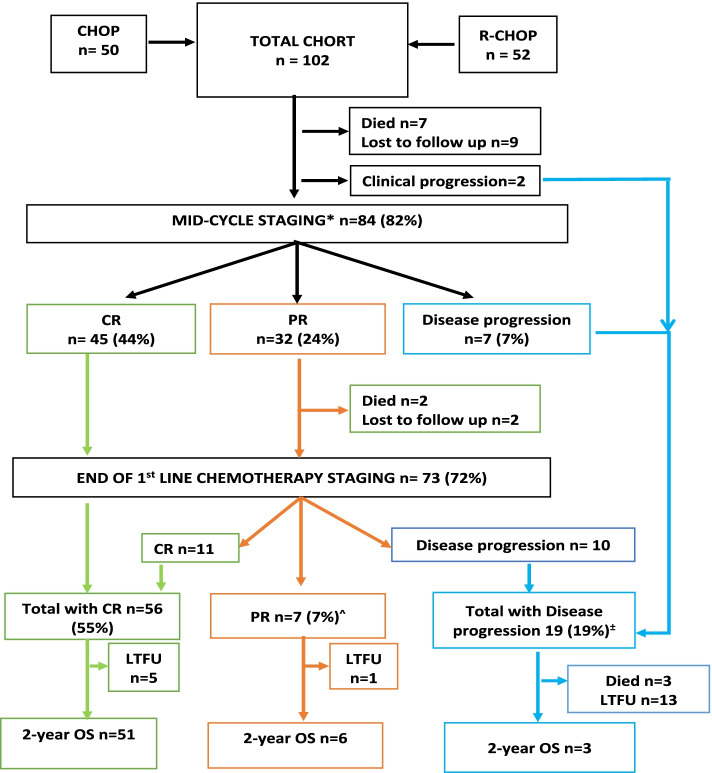
Treatment, response, and 2-year outcome in CD 20 positive HAL Abbreviations: *CHOP* cyclophosphamide, doxorubicin, vincristine, prednisone, *R* rituximab, *CR* complete response, *PR* partial response, *LTFU *lost to follow up, *OS* overall survival. *10 patients had CT scans and 74 patients had PET-CT scans at mid-cycle re-staging. ^^^6 patients received 2^nd^ line chemotherapy and/or radiotherapy. ^±^10 patients received 2^nd^ line chemotherapy

Fifty patients received CHOP and 52 R-CHOP, with an overall CR in 56 (55%) (95% CI 45%; 65%). All 45 patients who achieved a mid-cycle CR remained in CR at the end of first-line chemotherapy (Fig. 1). Of the nine patients with progressive disease at mid-cycle, 2 patients received 2nd-line chemotherapy and were then lost to follow-up within 6 months. Three of these 9 patients demised and another 4 were lost to follow-up (3 within 6 months and the 9th patient defaulted therapy at 14 months).

**Fig. 2:**
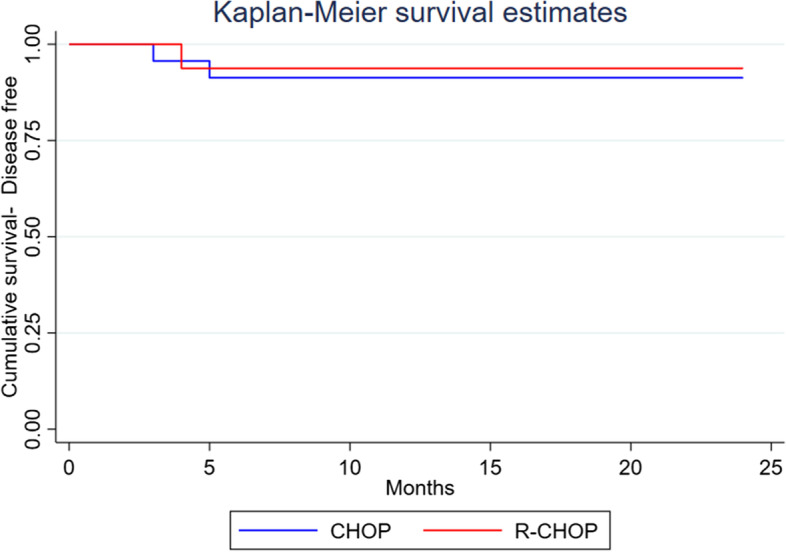
Disease-free survival by group Abbreviations: *CHOP* cyclophosphamide, doxorubicin, vincristine, prednisone, *R* rituximab

**Fig. 3:**
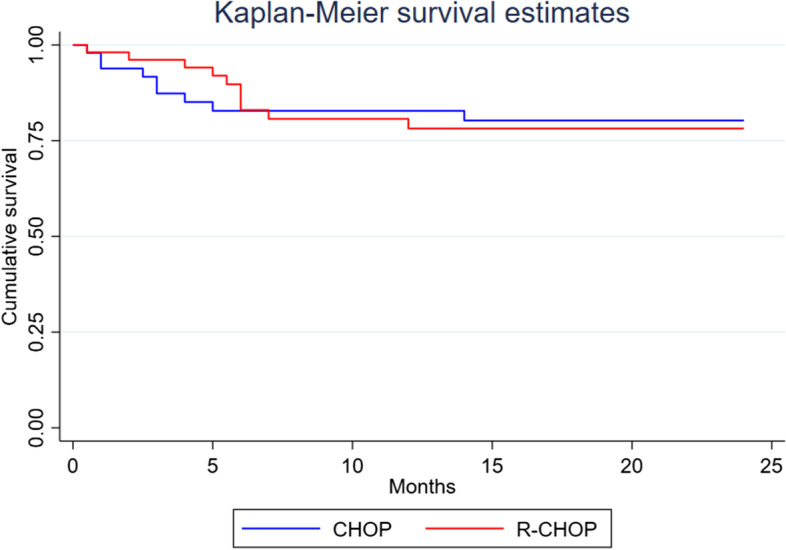
Overall survival at 2 years by group Abbreviations: *CHOP* cyclophosphamide, doxorubicin, vincristine, prednisone, *R* rituximab

Twenty-three patients (46%) receiving CHOP achieved a CR (95% CI 32%; 61%) and 33(64%) on R-CHOP attained a CR (95% CI 49%; 76%). At the end of first-line chemotherapy, 56 patients were in a CR (45 patients at mid-cycle and 11 patients converted from mid-cycle PR to end-of-treatment CR). Two patients with a CR relapsed at 4 and 5 months. Both patients received salvage chemotherapy, with 1 achieving a second CR with salvage chemotherapy and remaining in CR at 2 years and the second patient defaulted follow-up after 12 months. The disease-free survival and overall survival for the 2 treatment groups are shown in Figs. 2 and 3, respectively. Overall, 12 patients died during treatment. Nine patients died before completing chemotherapy and 3 patients died after being re-staged and assessed as having progressive disease. Eleven patients died within 6 months and the twelfth patient died at 10 months with sepsis. Sepsis accounted for four deaths (8%) in the CHOP arm and two (4%) in the R-CHOP group. The remaining six deaths were attributed to the progression of lymphoma (4 patients) or toxicity of chemotherapy or lymphoma (2 patients). The results are described in Table 2.

**Table 2:** Response to first-line chemotherapy for patients with CD20-positive HAL treated at King Edward VIII Hospital

		Total ***n*** = 102 (%)	CHOP ***n*** = 50 (%)	R-CHOP ***n*** = 52 (%)	***P*** value (CHOP vs R-CHOP)
**Complete response**		56 (55%)	23 (46%)	33 (64%)	0.147
**Total no. of chemotherapy cycles received by 56 patients that achieved CR**	**3–5**	4 (7.2%)	1 (4.3%)	3 (9.1%)	0.240
	**6–8**	52 (92.8%)	22 (95.7%)	30 (90.9%)	
**Partial response**		7 (7%)	6 (12%)	1 (2%)	0.220
No response/progressive disease*		14 (14%)	7 (14%)	7 (14%)	
Lost to follow-up**		13 (13%)	7 (14%)	6 (12%)	
**Died**^**#**^		12 (12%)	7 (14%)	5 (10%)	
**2-year DFS**		47 (46%)	21 (42%)	26 (50%)	0.585
**2-year OS**		60 (59%)	30 (60%)	30 (58%)	0.100
**Concurrent ART**		86 (84%)	38 (76%)	48 (92%)	0.027
**Hospitalization**		29 (28%)	12 (24%)	17 (33%)	0.331
**Infections±**		29 (28%)	13 (26%)	16 (31%)	0.594
**Infectious complications CD4 > 50 cells/μL (*n*** **= 91)**		25 (28%)	10/42 (23%)	15/49 (30%)	0.381
**Received red cell transfusion**		27 (26%)	9 (18%)	18 (35%)	0.057
**Received granulocyte colony-stimulating factor**		35 (34%)	17 (34%)	18 (35%)	0.915

*HAL* HIV-associated lymphoma, *CHOP* cyclophosphamide, adriamycin, vincristine, prednisone, *R-CHOP* rituximab with CHOP, *CR* complete response, ±Excluding TB, *DFS* disease-free survival, *OS* overall survival, *ART* anti-retroviral therapy

*An additional 5 patients with disease progression were lost to follow-up or demised

**2 patients had progressive disease at the time of loss of follow-up

^#^3 patients had progressive disease at the time of death

### Infectious complications

The overall incidence of infectious complications was similar in the two treatment arms (Table 2). Eleven patients were on TB treatment at diagnosis of lymphoma, seven received CHOP and four R-CHOP. Two were diagnosed with TB based on findings on the initial staging bone marrow examination. One had granulomatous inflammation consistent with TB with no acid-fast bacilli seen or cultured. In the other bone marrow, acid-fast bacilli were seen and *Mycobacterium tuberculosis* was cultured. During chemotherapy, six patients on CHOP and three on R-CHOP developed TB, eight pulmonary, and one both pulmonary and meningeal TB. None of the patients on CHOP received TB chemoprophylaxis while 69% on R-CHOP did.

Of 13 infections in the CHOP group, seven led to admission and were due to *Klebsiella septicemia*, TB meningitis, cellulitis, cryptococcal meningitis, lung abscess, bronchitis, and neutropenic sepsis. Eight of 16 infections in the R-CHOP group led to admission. One had sepsis at the site of tumor biopsy, four had neutropenic sepsis, two had pneumonia and one had shingles. Infections managed as outpatients involved the upper respiratory, lower respiratory, and gastrointestinal tracts. In the subgroup with CD4 cell count < 50 cells/μl, three of eight in the CHOP and one of three in the R-CHOP group developed sepsis. All of the former died from sepsis. Overall in the whole cohort, infections were seen in 36% of patients with CD4 < 50 cells/μl, and 28% with CD4 > 50 cells/μl (*p*=0.580).

### CNS disease

Seven patients had CNS disease at presentation, four with leptomeningeal and three with parenchymal involvement. Of the four that presented with leptomeningeal disease, three were treated with CHOP and triple IT chemotherapy. One attained CR and survived 72 months. The other two had leptomeningeal relapse within a year of presentation; one achieved a CR with high dose methotrexate (HD-MTX) and survived 71 months, then defaulted and the other also defaulted treatment with OS of 14 months. The remaining patient presenting with leptomeningeal disease, cleared the CNS disease after completing six cycles of R-CHOP and triple IT chemotherapy but was lost to follow-up after eight months. An additional patient was noted to have leptomeningeal involvement after two cycles of R-CHOP. This patient died after receiving four more cycles of R-CHOP and three doses of IT chemotherapy.

All three patients with parenchymal disease received HD-MTX. The one patient that received CHOP and two cycles of HD-MTX attained a PR and was lost to follow-up after 27 months. Of the two that received R-CHOP, one was lost to follow-up after six cycles of chemotherapy and the other achieved a CR and survived 30 months.

## Discussion

This is the first study from KZN, South Africa, to describe the profile and outcomes of CD20-positive HAL. The median age of the cohort was 39 years, 54% females, and approximately 50% with advanced-stage disease and high-risk International Prognostic Index scores. This is similar to cohorts described in Europe and Asia with the exception of the sex distribution [27–29]. From 2008 to 2013, during which time 49% of the cohort had already presented, the standard of treatment was CHOP chemotherapy. The female predominance during that period is best explained by the demographic profile of HIV in SA [30, 31], which is consistent with other reports from SA [32, 33]. From 2013 onwards the treatment of choice was R-CHOP. The male predominance during this period was likely due to a combination of improved uptake of ART by males as the rollout of ART in SA matured and a shift in health-seeking behavior of males from traditional to conventional medical care [34].

HAL is associated with more extranodal disease and bone marrow involvement [35, 36]. Here, consistent with other reports, more than 70% of patients presented with extranodal disease [36]. The 5% bone marrow involvement contrasts with other studies from SA and Europe where marrow involvement ranged from 13 to 35% [33, 37]. It is noteworthy that in this clinical cohort bone marrow involvement was determined exclusively by histology, without the use of the more sensitive routine immunohistochemical and immunophenotyping techniques currently available [38].

The complete response of 55% (95% CI 45%; 65%) and 2-year OS of 59% (95% CI 50%, 69%) was an improvement on a study from the Western Cape province of SA which reported a CR of 39% and 2 year OS of 40.5% [14]. Of interest a study from Uganda, which like the Western Cape study reported exclusively on the use of CHOP, showed a CR of 27% [14, 39].

As rituximab became available in 2013, a change in treatment regimen halfway through the study to include rituximab created an opportunity to report on the response to rituximab. The CR increased by 18% with rituximab. These results are similar to the first randomized trial comparing CHOP and R-CHOP by Kaplan et al. [40]. It is worth noting that the benefit of rituximab internationally was only demonstrated when individual patient-level data was pooled to achieve adequate power for analysis [10, 41]. Of interest, the CR and OS with R-CHOP here were similar to that described in studies from Europe [36, 40].

Case reports of rituximab use in rheumatology practice have shown an increased incidence of TB. In this study, rituximab did not increase the incidence of TB, which is consistent with reports on its use in hematology patients in general [42–44]. However, when interpreting this one should take into account that Isoniazid prophylactic therapy was taken by 69% of patients receiving R-CHOP and none that were taking CHOP, which might have biased the outcome. British and European guidelines do not recommend TB prophylaxis with rituximab, even in high prevalence settings which is our current practice [45].

Anemia is an established side effect of rituximab, but poorly described in this context [40]. Here, there was a trend towards increased transfusion requirements among patients receiving rituximab. A possible confounder was the increased use of ART with R-CHOP which might have contributed to the anemia [46].

Due to multiple challenges, including patient and health care factors, routine CNS prophylaxis was not practiced. In resource-limited settings, a more cost-effective and pragmatic approach is favored. This includes high vigilance for clinical signs of CNS disease and a low threshold to perform imaging or spinal fluid examination. Interestingly this strategy detected a comparable proportion of CNS disease as that reported in a UK cohort with routine CNS cytology [47], even though it is generally accepted that up to 25% of CNS involvement may be clinically silent [48]. Our findings compare with Barta et al. who described a 5% risk of CNS relapse and 50% 2-year progression-free survival; with our OS exceeding 24 months for the three patients that attained CR [49]. Although numbers are small the findings here suggest a clinically based approach to CNS involvement is not unreasonable.

### Limitations of the study

The retrospective study design hampered the availability of, and access to, clinical and laboratory data. Red cell transfusion records prior to referral to King Edward VIII Hospital were not available for most patients, and the difference in baseline hemoglobin between treatment groups could not be accurately determined. Biopsy and bone marrow examinations were reported on by different pathologists possibly introducing observer bias. A serious limiting factor in comparing outcomes between the two treatment groups was the size of the groups. To see a statistically different CR of 10%, a sample size of 388 patients per group was needed based on a test of equality [50].

## Conclusion

The clinical characteristics of patients presenting with HAL in a high HIV prevalence, the resource-limited setting was no different to cohorts described elsewhere except for a preponderance of females and a lower incidence of bone marrow involvement. Overall treatment outcomes were very similar to other cohorts despite the unique socio-economic and healthcare challenges.

## Data Availability

All patient files are stored in the hematology clinic filing room at King Edward VIII Hospital and are available for review.

## Abbreviations

HIV: Human immunodeficiency virus
HAL: HIV-associated lymphoma
R: Rituximab
CR: Complete response
ART: Antiretroviral therapy
DFS: Disease-free survival
OS: Overall survival
CHOP: Cyclophosphamide, adriamycin, vincristine, prednisone
SA: South Africa
CT: Computerized tomography
PET: Positron emission tomography
CNS: Central nervous system
CSF: Cerebrospinal fluid
IT: Intrathecal
TB: Tuberculosis

## References

[1] Lewden C, May T, Rosenthal E, Burty C, Bonnet F, Costagliola D, et al. Changes in causes of death among adults infected by HIV between 2000 and 2005: the “Mortalité 2000 and 2005” surveys. J Acquir Immune Defic Syndr. 2008;48(5):590–8. 18645512 10.1097/QAI.0b013e31817efb54

[2] Schneider E, Whitmore S, Glynn KM, Dominguez K, Mitsch A, McKenna MT. Revised surveillance case definitions for HIV infection among adults, adolescents and children aged <18 months and for HIV infection and AIDS among children aged 18 months to <13 years--United States, 2008. MMWR Recomm Rep. 2008;57(RR-10):1–12. 19052530

[3] Seaberg EC, Wiley D, Martinez-Maza O, Chmiel JS, Kingsley L, Tang Y, et al. Cancer incidence in the multicenter AIDS Cohort Study before and during the HAART era: 1984-2007. Cancer. 2010;116(23):5507–16. 20672354 10.1002/cncr.25530 PMC2991510

[4] South African National AIDS Council. Let our actions count: South Africa’s National Strategic Plan on HIV, TB and STIs 2017-2022. [homepage on the internet]. No date Available from :https://sanac.org.za/wp-content/uploads/2018/09/NSP_FullDocument_FINAL.pdf.

[5] Statistics South Africa 2019: Mid-year population estimates by ISI House [homepage on the internet] No date Available from:https://www.statssa.gov.za/publications/P0302/P03022019.pdf

[6] Barta SK, Dunleavy K, Mounier N. Diffuse large B cell lymphoma. HIV-associated haematological malignancies. Springer International Publishing;2016.

[7] Weiss R, Mitrou P, Arasteh K, Schuermann D, Hentrich M, Duehrsen U, et al. Acquired immunodeficiency syndrome-related lymphoma: Simultaneous treatment with combined cyclophosphamide, doxorubicin, vincristine, and prednisone chemotherapy and highly active antiretroviral therapy is safe and improves survival—Results of the German Multicenter Trial. Cancer. 2006;106(7):1560–8. 16502436 10.1002/cncr.21759

[8] Mounier N, Spina M, Gabarre J, Raphael M, Rizzardini G, Golfier JB, et al. AIDS-related non-Hodgkin lymphoma: final analysis of 485 patients treated with risk-adapted intensive chemotherapy. Blood. 2006;107(10):3832–40. 16410446 10.1182/blood-2005-09-3600

[9] Coiffier B, Lepage E, Brière J, Herbrecht R, Tilly H, Bouabdallah R, et al. CHOP chemotherapy plus rituximab compared with CHOP alone in elderly patients with diffuse large-B-cell lymphoma. New England Journal of Medicine. 2002;346(4):235–42. 11807147 10.1056/NEJMoa011795

[10] Barta SK, Xue X, Wang D, Tamari R, Lee JY, Mounier N, et al. Treatment factors affecting outcomes in HIV-associated non-Hodgkin lymphomas: a pooled analysis of 1546 patients. Blood. 2013;122(19):3251–62. 24014242 10.1182/blood-2013-04-498964 PMC3821722

[11] Brunnberg U, Hentrich M, Hoffmann C, Wolf T, Hübel K. HIV-associated malignant lymphoma. Oncol Res Treat. 2017;24(40):82–7. 10.1159/000456036 28253516

[12] Meister A, Hentrich M, Wyen C, Hübel K. Malignant lymphoma in the HIV-positive patient. Eur J Haematol. 2018;101(1):119–26. 29663523 10.1111/ejh.13082

[13] Hentrich M, Hoffmann C, Mosthaf F, Müller M, Siehl J, Wyen C, et al. Therapy of HIV-associated lymphoma-recommendations of the oncology working group of the German study group of physicians in private practice treating HIV-infected patients (DAGNÄ), in cooperation with the German AIDS Society (DAIG). Ann Hematol. 2014;93(6):913–21. 24807241 10.1007/s00277-014-2058-4

[14] De Wit P, Maartens DJ, Uldrich TS, Sissolak G. Treatment outcomes in AIDS-related diffuse large B-cell lymphoma in the setting roll out of combination antiretroviral therapy in South Africa. J Acquir Immune Defic Syndr. 2013;64(1):66–73. 23797692 10.1097/QAI.0b013e3182a03e9b PMC3797444

[15] Campo E, Swerdlow SH, Harris NL, Pileri S, Stein H, Jaffe ES. The 2008 WHO classification of lymphoid neoplasms and beyond: evolving concepts and practical applications. Blood. 2011;117(19):5019–32. 21300984 10.1182/blood-2011-01-293050 PMC3109529

[16] Jaffe ES, Harris NL, Stein H, Vardiman JW. Classification of tumours: pathology and genetics of tumours of haemopoeitic and lymphoid tissues (World Health Organisation). Lyon: IARC Press; 2001.

[17] Shipp MA, Harrington DP, Anderson JR, Armitage JO, Bonadonna G, Brittinger G, et al. A predictive model for aggressive non-Hodgkin’s lymphoma. The International Non-Hodgkin’s Lymphoma Prognostic Factors Project. N Engl J Med. 1993;329(14):987–94. 10.1056/NEJM199309303291402. 8141877 10.1056/NEJM199309303291402

[18] Lister TA, Crowther D, Sutcliffe SB, Glatstein E, Canellos GP, Young RC, et al. Report of a committee convened to discuss the evaluation and staging of patients with Hodgkin’s disease: Cotswolds meeting. J Clin Oncol. 1989;7(11):1630–6. 2809679 10.1200/JCO.1989.7.11.1630

[19] Meintjes G, Moorhouse MA, Carmona S, Davies N, Dlamini S, Van Vuuren C, et al. Adult antiretroviral therapy guidelines 2017. Southern Afr J HIV Med. 2017;18(1):1–24. 10.4102/sajhivmed.v18i1.776 PMC5843236 29568644

[20] Fisher RI, Gaynor ER, Dahlberg S, Oken MM, Grogan TM, Mize EM, et al. Comparison of a standard regimen (CHOP) with three intensive chemotherapy regimens for advanced non-Hodgkin’s lymphoma. New England Journal of Medicine. 1993;328(14):1002–6. 7680764 10.1056/NEJM199304083281404

[21] Tilly H, Vitolo U, Walewski J, da Silva MG, Shpilberg O, Andre M, et al. Diffuse large B-cell lymphoma (DLBCL): ESMO Clinical Practice Guidelines for diagnosis, treatment and follow-up. Ann Oncol. 2012;23(7):78–82. 22997459 10.1093/annonc/mds273

[22] Kridel R, Dieterich PY. Prevention of CNS relapse in diffuse large B-cell lymphoma. Lancet Oncol. 2011;12(13):1258–66. 21933751 10.1016/S1470-2045(11)70140-1

[23] Lyman GH. Guidelines of the National Comprehensive Cancer Network on the use of myeloid growth factors with cancer chemotherapy: a review of the evidence. JNatl Compr Cancer Netw. 2005;3(4):557–71. 10.6004/jnccn.2005.0031 16038646

[24] Meignan M, Gallamini A, Haioun C. Report on the First International Workshop on Interim-PET- Scan in Lymphoma. Leuk Lymphoma. 2009;50(8):1257–60. 19544140 10.1080/10428190903040048

[25] Cheson BD, Pfistner B, Juweid ME, Gascoyne RD, Specht L, Horning SJ, et al. Revised response criteria for malignant lymphoma. Journal of clinical oncology. 2007;25(5):579–86. 17242396 10.1200/JCO.2006.09.2403

[26] Barrington SF, Mikhaeel NG. When should FDG-PET be used in the modern management of lymphoma? Br J Haematol. 2014;164(3):315–28. 24131306 10.1111/bjh.12601

[27] Adam J, Olszewski MD, Jaleh Fallah MD, Jorge J, Castillo MD. Human immunodeficiency virus-associated lymphomas in the antiretroviral therapy era: analysis of the National Cancer Data Base. Cancer. 2016;122(17):2689–97. 27337679 10.1002/cncr.30112

[28] Sampath R, Manipadam MT, Nair S, Viswabandya A, Zachariah A. HIV-associated lymphoma: a 5-year clinicopathologic study from India. Ind J Pathol Microbiol. 2019;62(1):73–8. 10.4103/IJPM.IJPM_452_17 30706863

[29] Gabarre J, Raphael M, Lepage E, Martin A, Oksenhendler E, Xerri L, et al. Human immunodeficiency virus–related lymphoma: relation between clinical features and histologic subtypes. The American Journal of medicine. 2001;111(9):704–11. 11747850 10.1016/s0002-9343(01)01020-8

[30] Shisana O, Simbayi L. Nelson Mandela HSRC study of HIV/AIDS. South African National HIV prevalence, behavioural risks and mass media. Household survey 2002. Pretoria: HSRC; 2002.

[31] Shisana O, Rehle T, Simbayi LC, Zuma K, Jooste S, Zungu N, et al. South African National HIV prevalence, incidence and behavior survey, 2012. Cape Town: HSRC Press; 2014. 10.2989/16085906.2016.1153491 27002359

[32] Patel M, Philip V, Omar T, Turton D, Candy G, Lakha A, et al. The impact of human immunodeficiency virus infection (HIV) on lymphoma in South Africa. J Cancer Ther. 2015;(6):527–35.

[33] Magangane PS, Mohamed Z, Naidoo R. Diffuse large B cell lymphoma in a high human immunodeficiency virus (HIV) prevalence, low-resource setting. S. Afr. J. Oncol. 2020;4(0):a104.

[34] Cooke GS, Tanser FC, Bärnighausen TW, Newell ML. Population uptake of antiretroviral treatment through primary care in rural South Africa. BMC Public Health. 2010;10(1):1–9. 20920267 10.1186/1471-2458-10-585 PMC3091553

[35] Ribera JM, Oriol A, Morgades M, González-Barca E, Miralles P, Lopez-Guillermo A, et al. Safety and efficacy of cyclophosphamide, adriamycin, vincristine, prednisone and rituximab in patients with human immunodeficiency virus-associated diffuse large B-cell lymphoma: results of a phase 11 trial. Br J Haematol. 2008;140(4):411–9. 18162120 10.1111/j.1365-2141.2007.06943.x

[36] Besson C, Lancar R, Prevot S, Algarte-Genin M, Delobel P, Bonnet F, et al. Outcomes for HIV-associated diffuse large B-cell lymphoma in the modern combined antiretroviral therapy era. Aids. 2017;31(18):2493–501. 28926410 10.1097/QAD.0000000000001652

[37] Besson C, Goubar A, Gabarre J, Rozenbaum W, Pialoux G, Châtelet FP, et al. Changes in AIDS-related lymphoma since the era of highly active antiretroviral therapy. Blood. 2001;98(8):2339–44. 10.1182/blood.v98.8.2339 PMID: 11588028. 11588028 10.1182/blood.v98.8.2339

[38] Kremer M, Quintanilla-Martinez L, Nahrig J, von Schilling C, Fend F. Immunohistochemistry in bone marrow pathology: a useful adjunct for morphologic diagnosis. Virchows Arch. 2005;447:920–37. 16231177 10.1007/s00428-005-0070-8

[39] Okello CD, Omoding A, Ddungu H, Mulumba Y, Orem J. Outcomes of treatment with CHOP and EPOCH in patients with HIV associated NHL in a low resource setting. BMC Cancer. 2020;20(1):1–9. 10.1186/s12885-020-07305-2 PMC7446121 32831073

[40] Kaplan LD, Lee JY, Ambinder RF, Sparano JA, Cesarman E, Chadburn A, et al. Rituximab does not improve clinical outcome in a randomised phase 3 trial of CHOP with or without Rituximab in patients with HIV-associated non-Hodgkin lymphoma: AIDS Malignancies Consortium Trial 010. Blood. 2005;106(5):1538–43. 15914552 10.1182/blood-2005-04-1437 PMC1895225

[41] Castillo JJ, Echenique IA. Rituximab in combination with chemotherapy versus chemotherapy alone in HIV-associated non-Hodgkin lymphoma: a pooled analysis of 15 prospective studies. Am J Hematol. 2012;87(3):330-3 10.1002/ajh.22275 22308010

[42] Loveday M, Mzobe YN, Pillay Y, Barron P. Figures of the dead: a decade of tuberculosis mortality registration in South Africa. South Afr Med J. 2019;109(10):728–32. 10.7196/SAMJ.2019.v109i10.14073 31635566

[43] Alkadi A, Alduaiji AA. Risk of tuberculosis reactivation with rituximab therapy. Int J Health Sci (Qassim). 2017;11(2):41–4. 28539862 PMC5426414

[44] Xiao J, Du S, Dai G, Gao G, Yang D, Zhao H. Efficacy and tolerability of chemotherapy in Chinese patients with AIDS-related Burkitt lymphoma and diffuse large B-cell lymphoma: an observational study. Sci Rep. 2017;7(1):1–8. 28507339 10.1038/s41598-017-02086-4 PMC5432515

[45] Mikulska M, Lanini S, Gudiol C, Drgona L, Ippolito G, Fernandez-Ruiz M, et al. ESCMID Study Group for Infections in Compromised Hosts (ESGICH) Consensus Document on the safety of targeted and biological therapies: an infectious diseases perspective (agents targeting lymphoid cells surface antigens [I]: CD19, CD20 and CD52). Clin Micropbio Infection Feb. 2018;24:S71–82. 10.1016/j.cmi.2018.02.003 29447988

[46] Mounier N, Rudek MA. Chemotherapy and interactions with combination antiretroviral therapy. In HIV-associated hematological malignancies 2016 (pp. 207-214). Springer, Cham.

[47] Sarker D, Thirwell C, Nelson M, Gazzard B, Bower M. Leptomeningeal disease in AIDS-related non-Hodgkin’s lymphoma. AIDS. 2003;17(6):861–5. 12660533 10.1097/00002030-200304110-00011

[48] Straus DJ. Human immunodeficiency virus-associated lymphomas. Med Clin North Am. 1997;81(2):495–510. 9093239 10.1016/s0025-7125(05)70528-9

[49] Barta SK, Joshi J, Mounier N, Xue X, Wang D, Ribera JM, et al. Central nervous system involvement in AIDS-related lymphomas. Brit J Haematol. 2016;173(6):857–66. 27062389 10.1111/bjh.13998 PMC4900917

[50] Wang H, Chow S-C. Sample size calculation for comparing proportions. Wiley Encyclopedia of Clinical Trials; 2007.

